# Regional antibiotic delivery for sternal wound infection prophylaxis a systematic review and meta-analysis of randomized controlled trials

**DOI:** 10.1038/s41598-024-60242-z

**Published:** 2024-04-27

**Authors:** Mariusz Kowalewski, Michalina M. Kołodziejczak, Tomasz Urbanowicz, Maria Elena De Piero, Silvia Mariani, Michał Pasierski, Maged Makhoul, Maria Comanici, Emil Julian Dąbrowski, Matteo Matteucci, Giulio Massimi, Radosław Litwinowicz, Adam Kowalówka, Wojciech Wańha, Federica Jiritano, Gennaro Martucci, Giuseppe Maria Raffa, Pietro Giorgio Malvindi, Łukasz Kuźma, Piotr Suwalski, Roberto Lorusso, Paolo Meani, Harold Lazar, Thoracic Research Centre, Jakub Brączkowski, Dario Fina, Mirosław Gozdek, Giovanni Chiarini, Federica Jiritano, Michalina M. Kołodziejczak, Adam Kowalówka, Mariusz Kowalewski, Łukasz Kuźma, Roberto Lorusso, Radosław Litwinowicz, Tong Li, Giuseppe Marchese, Gennaro Martucci, Giulio Massimi, Matteo Matteucci, Maged Makhoul, Pietro Giorgio Malvindi, Silvia Mariani, Paolo Meani, Anna Olasińska, Michał Pasierski, Luigi Pannone, Maria Elena De Piero, Giuseppe Maria Raffa, Sebastian Stec, Jakub Staromłyński, Serena Todaro, Tomasz Urbanowicz, Wojciech Wańha

**Affiliations:** 1 Clinical Department of Cardiac Surgery and Transplantology, National Medical Institute of the Ministry of Interior and Administration, Wołoska 137, 02-507 Warsaw, Poland; 2 Cardio-Thoracic Surgery Department, Heart and Vascular Centre, Maastricht University Medical Centre, Cardiovascular Research Institute Maastricht (CARIM), Maastricht, The Netherlands; 3 Department of Anaesthesiology and Intensive Care, Antoni Jurasz University Hospital No. 1, Collegium Medicum Nicolaus Copernicus University, Bydgoszcz, Poland; 4 Cardiac Surgery and Transplantology Department, Poznań University of Medical Sciences, Poznan, Poland; 5 Department of Cardiac Surgery, Harefield Hospital, London, UK; 6 Department of Invasive Cardiology, Medical University of Bialystok, Bialystok, Poland; 7 Cardiac Surgery Unit, Department of Medicine and Surgery, ASST dei Sette Laghi, University of Insubria, Varese, Italy; 8 Cardiac Surgery Unit, Santa Maria della Misericordia Hospital, Perugia, Italy; 9 Department of Cardiac Surgery, Regional Specialist Hospital, Grudziądz, Poland; 10 Department of Cardiac Surgery, Faculty of Medical Sciences, Upper-Silesian Heart Center, Medical University of Silesia, Katowice, Poland; 11 Department of Invasive Cardiology, School of Medicine in Katowice, Medical University of Silesia, Katowice, Poland; 12 Department of Experimental and Clinical Medicine, Magna Graecia University, Catanzaro, Italy; 13 Department of Anesthesia and Intensive Care, Istituto Mediterraneo Per i trapianti e Terapie ad alta specializzazione (IRCCS-ISMETT), Palermo, Italy; 14 Department for the Treatment and Study of Cardiothoracic Diseases and Cardiothoracic Transplantation, IRCCS-ISMETT, Palermo, Italy; 15 Cardiac Surgery Unit, Lancisi Cardiovascular Center, Ospedali Riuniti Delle Marche, Polytechnic University of Marche, Ancona, Italy; 16 Department of Cardiothoracic and Vascular Anesthesia and Intensive Care Unit, IRCCS Policlinico, San Donato Milanese, Milan, Italy; 17 Boston University School of Medicine, Boston, MA USA; 18 Thoracic Research Centre, Innovative Medical Forum, Collegium Medicum Nicolaus Copernicus University, Bydgoszcz, Poland; 19 Department of Cardiology, Città di Lecce Hospital, GVM Care and Research, Lecce, Italy; 20 Department of Cardiology, Hospital of the Ministry of Interior, Bydgoszcz, Poland; 21 Division of Anesthesiology, Intensive Care and Emergency Medicine, University of Brescia, Brescia, Italy; 22 Department of Experimental and Clinical Medicine, “Magna Graecia” University of Catanzaro, Catanzaro, Italy; 23 Department of Anesthesiology and Intensive Care, Collegium Medicum Bydgoszcz, Antoni Jurasz University Hospital No.1, Nicolaus Copernicus University Torun, 85-094 Bydgoszcz, Poland; 24 Department of Cardiac Surgery, School of Medicine in Katowice, Medical University of Silesia, Katowice, Poland; 25 Department of Cardiac Surgery, Upper-Silesian Heart Center, Katowice, Poland; 26 Cardiovascular Research Institute Maastricht (CARIM), Maastricht, The Netherlands; 27 CAROL - Cardiothoracic Anatomy Research Operative Lab, Department of Cardiovascular Surgery and Transplantology, Institute of Cardiology, Jagiellonian University Medical College, Krakow, Poland; 28 Department of Cardiothoracic, Transplantation and Vascular Surgery, Hannover Medical School, Hannover, Germany; 29 Department of Pathophysiology and Transplantation, Università degli Studi di Milano, Milan, Italy; 30 Department of Anesthesia and Intensive Care, Istituto Mediterraneo per i Trapianti e Terapie ad alta Specializzazione (IRCCS-ISMETT), UPMCI (University of Pittsburgh Medical Center Italy), Palermo, Italy; 31 Department of Medicine and Surgery, Circolo Hospital, University of Insubria, Varese, Italy; 32 Heart Rhythm Management Centre, Postgraduate Program in Cardiac Electrophysiology and Pacing, European Reference Networks Guard-Heart, Universitair Ziekenhuis Brussel - Vrije Universiteit Brussel, Brussels, Belgium; 33 Division of Electrophysiology, Cardioneuroablation, Catheter Ablation and Cardiac Stimulation, Subcarpathian Center for Cardiovascular Intervention, Sanok, Poland; 34 Department of Cardiology and Structural Heart Diseases, Medical University of Silesia, Katowice, Poland

**Keywords:** Regional antibiotic delivery, Sternal wound, Cardiac surgery, Mediastinitis, Interventional cardiology, Antimicrobial resistance, Clinical microbiology, Microbiology, Cardiology, Risk factors

## Abstract

Despite evidence suggesting the benefit of prophylactic regional antibiotic delivery (RAD) to sternal edges during cardiac surgery, it is seldom performed in clinical practice. The value of topical vancomycin and gentamicin for sternal wound infections (SWI) prophylaxis was further questioned by recent studies including randomized controlled trials (RCTs). The aim of this systematic review and meta-analysis was to comprehensively assess the safety and effectiveness of RAD to reduce the risk of SWI.We screened multiple databases for RCTs assessing the effectiveness of RAD (vancomycin, gentamicin) in SWI prophylaxis. Random effects meta-analysis was performed. The primary endpoint was any SWI; other wound complications were also analysed. Odds Ratios served as the primary statistical analyses. Trial sequential analysis (TSA) was performed.Thirteen RCTs (N = 7,719 patients) were included. The odds of any SWI were significantly reduced by over 50% with any RAD: OR (95%CIs): 0.49 (0.35–0.68); *p* < 0.001 and consistently reduced in vancomycin (0.34 [0.18–0.64]; *p* < 0.001) and gentamicin (0.58 [0.39–0.86]; *p* = 0.007) groups (*p*_subgroup_ = 0.15). Similarly, RAD reduced the odds of SWI in diabetic and non-diabetic patients (0.46 [0.32–0.65]; *p* < 0.001 and 0.60 [0.44–0.83]; *p* = 0.002 respectively). Cumulative Z-curve passed the TSA-adjusted boundary for SWIs suggesting adequate power has been met and no further trials are needed. RAD significantly reduced deep (0.60 [0.43–0.83]; *p* = 0.003) and superficial SWIs (0.54 [0.32–0.91]; *p* = 0.02). No differences were seen in mediastinitis and mortality, however, limited number of studies assessed these endpoints. There was no evidence of systemic toxicity, sternal dehiscence and resistant strains emergence. Both vancomycin and gentamicin reduced the odds of cultures outside their respective serum concentrations’ activity: vancomycin against gram-negative strains: 0.20 (0.01–4.18) and gentamicin against gram-positive strains: 0.42 (0.28–0.62); *P* < 0.001. Regional antibiotic delivery is safe and effectively reduces the risk of SWI in cardiac surgery patients.

## Introduction

Sternal wound infections (SWIs) are among the most devastating complications following cardiac surgery and significantly increase postoperative morbidity and mortality^1^. Direct regional antibiotic delivery (RAD) to the sternal edges upon entering and just prior to closing the sternum, along with intravenous (iv) prophylactic antibiotics, has gained attention due to the potential in reducing surgical site infections (SSIs) following cardiac surgery^2^. Topical antibiotics, such as vancomycin or gentamicin, have been considered as a measure of antibiotic prophylaxis in cardiac surgery by the 2006 Society of Thoracic Surgeons (STS) Practice Guidelines (Class II, Level of Evidence B), which noted concerns about iv antibiotic penetration in the sternal area and the potential for infection with S. aureus^3^. As a result of additional studies showing the benefits of RAD in preventing SSIs, a class I recommendation (Level of Evidence B) was given in the 2016 prevention and management of sternal wound infections guidelines of the American Association for Thoracic Surgery (AATS) for the application of RAD to the cut edges of the sternum on opening and before closing in all cardiac surgical procedures involving a median sternotomy^1^. However, there were some concerns regarding potential elevated serum levels of RAD and the possibility of selecting antibiotic-resistant strains. In view of these concerns, the 2017 European Association for Cardio-Thoracic Surgery (EACTS) expert consensus highlighted the importance of careful monitoring and prudent use of this essential antibiotic^4^.

Controversies arose regarding RAD after recent randomized clinical trials (RCT) found that patients who were assigned to receive either vancomycin-soaked sponges or saline-soaked sponges had a similar occurrence of SWI (2.7% vs. 4.1%; *P* = 0.23)^5^. Therefore, the aim of this systematic review and meta-analysis of RCTs was to comprehensively assess the safety and effectiveness of RAD to reduce the risk of SWI in cardiac surgery procedures requiring a sternotomy.

## Methods

### Data sources and search strategy

Established methods were used in compliance with the updated Preferred Reporting Items for Systematic Reviews and Meta-Analyses (PRISMA) in the health care interventions statement^6^. A PRISMA checklist is available in Supplementary Table 1. We conducted a database screening for relevant studies up to May 16th 2023 through PubMed, EMBASE (Supplementary Table 2), the Cumulative Index of Nursing and Allied Health Literature (CINAHL), the Web of Science, the Cochrane Register of Controlled Clinical Trials, Clinical Key and Google Scholar registries, as well as published proceedings from major cardiac, thoracic, cardiothoracic, and cardiology society meetings. Abstracts were eligible for detailed assessment if available online and reporting outcomes of interest. Search terms included “vancom*cin; -paste, -gel, -ointment, -slurry”; “topical*-, local*-, regional*- vancom*cin”, gentam*cin, antiobiotic*; “vancom*cin/ gentam*cin/ antiobiotic AND stern*”; “vancom*cin/ gentam*cin/ antiobiotic AND mediastin*.” No language, publication date, or publication status restriction was imposed. Both blinded and open-label trials were considered eligible. The most updated or inclusive data for each study were used for abstraction. The references of original and review articles were cross-checked.

### Selection criteria and quality assessment

Studies were considered eligible when comparing prophylactic topically administered vancomycin- or gentamicin-based therapy versus no antibiotic or placebo in the setting of cardiac surgery performed via a median sternotomy. We restricted the search to these agents since these are endorsed in the guidelines^1^. Citations were screened at the title/abstract level and retrieved as full reports if they fulfilled the inclusion criteria: (1) human studies; (2) RCTs and (3) the reporting of a pre-specified outcome of SWI. We excluded studies which (1) were not of a randomized design; (2) reported no control group; (3) evaluated different regimens of RADs; (4) reported no clinical data. Studies in which a combination of different RADs were used and compared with no antibiotic or placebo were also considered for inclusion.

We extracted data for the included studies using a pre-specified datasheet. Variables in the pre-specified datasheet included study characteristics, demographic data, clinical characteristics, interventions, and outcomes.

Two independent reviewers (M.M.K. and M.P.) selected the studies for inclusion and extracted studies and patient characteristics of interest and relevant outcomes. Conflicts were resolved by consensus after discussion with a third reviewer (M.K.). Two authors (M.M.K. and M.P.) independently assessed the trials’ eligibility and risk of bias. The risk of bias for randomized studies was assessed using the components recommended by the Cochrane Collaboration^7^ including random sequence generation and random allocation; allocation concealment; blinding of participants, personnel, and outcome assessors; incomplete outcome data; selective outcome reporting; and other sources of bias^8^. Certainty of evidence was assessed by four main factors (risk of bias, inconsistency, indirectness, and imprecision) using the Grading of Recommendations Assessment, Development and Evaluations (GRADE) approach^9^. The certainty of the evidence was rated from high (ie, we are very confident that the true effect lies close to that of the effect estimate) to very low (ie, we have very little confidence in the effect estimate: the true effect is likely to be substantially different)^10^. Any discrepancies in bias assessment between the assessors were recorded.

### Outcome measures

The primary end point was the occurrence of any SWI in overall, diabetic and no-diabetic population. Secondary end points were the occurrence of deep SWI (DSWI), superficial SWI (SSWI), mediastinitis, and in-hospital mortality. Definitions for the type, degree, and depth of the infection were applied as per the study protocol. The review protocol was registered at PROSPERO database (nr CRD42022385529) and the current meta-analysis represents the portion of the protocol^11^.

### Statistical analysis

The analysis followed the intention-to-treat principle. Continuous variables were presented as mean and standard deviation (SD) for normally distributed data, while non-normally distributed variables were summarized as median and interquartile range (IQR). Group comparisons were conducted using the Mann–Whitney U test or appropriate standard t test. Odds ratios (ORs) with corresponding 95% confidence intervals (CIs) were calculated as summary statistics. Heterogeneity was evaluated using the Cochran Q test^12^ and the I^2^ statistic, with thresholds of 25%, 50%, and 75% representing low, moderate, and considerable degrees of heterogeneity, respectively^13^. Pooled ORs were computed using a random-effects model via the DerSimonian-Laird method, with the Mantel–Haenszel fixed-effects model utilized in case of moderate or low heterogeneity. Publication bias was explored for the primary endpoint using a funnel plot, assessed visually and through linear regression analysis^14^. To address studies reporting ‘0 events’, calculations were repeated using risk difference (RD) as the primary statistic. Bias risk was evaluated according to the Revised Cochrane risk-of-bias tool (RoB 2) (Supplementary Table 4). Sensitivity analyses were conducted by sequentially removing each study to assess its impact on the pooled results. Additionally, trial sequential analysis (TSA [Version 0.9.5.10 Beta, Copenhagen Trial Unit, Center for Clinical Intervention Research, Rigshospitalet, Copenhagen, Denmark]) was performed to validate the meta-analysis findings for SWI, maintaining a 5% type I error and 80% power. Review Manager 5.4 (The Nordic Cochrane Center, Copenhagen, Denmark) was employed for all analyses..

## Results

### Studies selection and patients baseline characteristics

**Figure 1:**
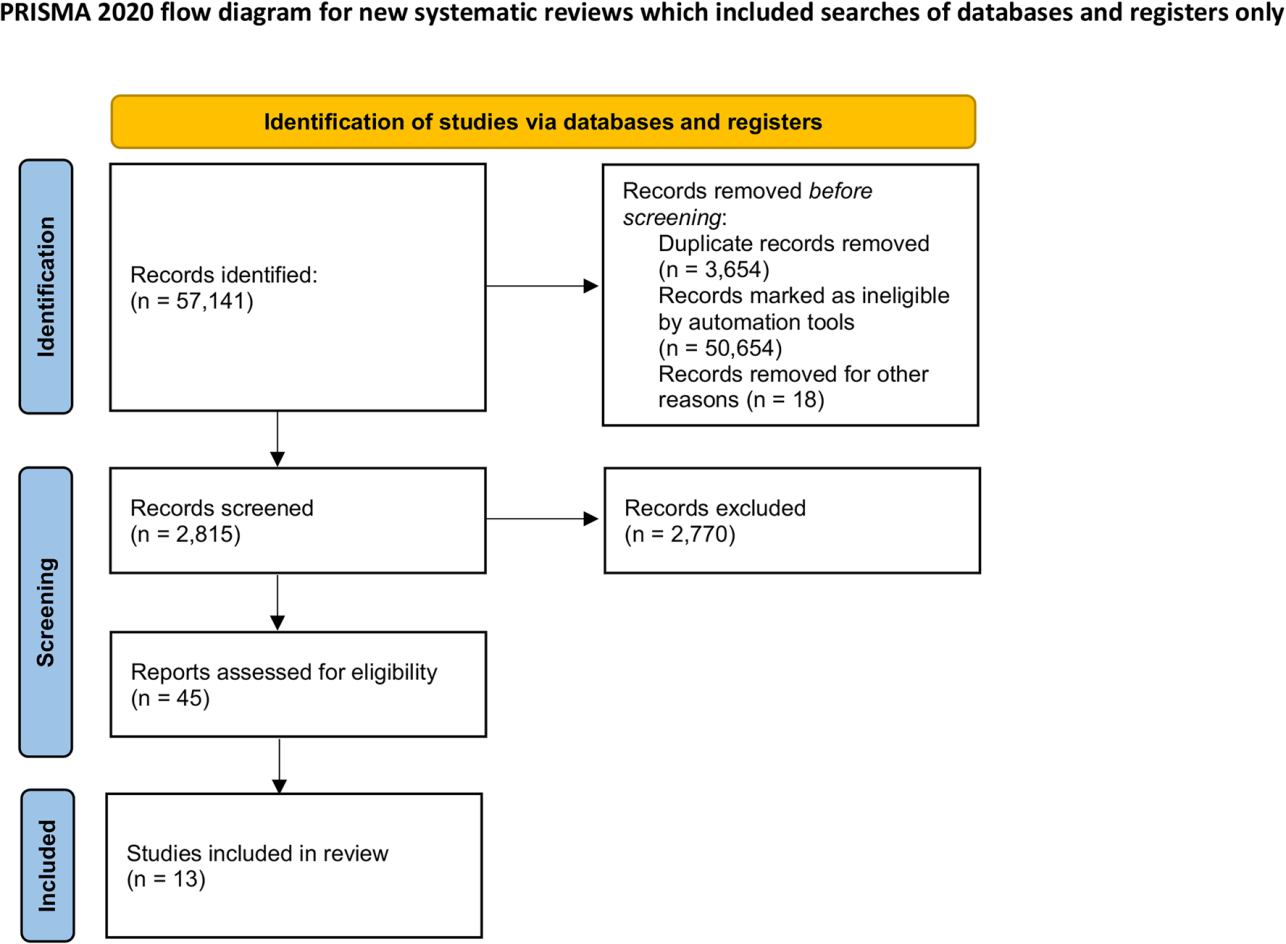
PRISMA flow diagram.

The current systematic review follows the GRADE criteria (Supplementary Table 3). Figure 1 depicts the process of study selection. Thirteen RCTs (7,719 patients) were included in the analysis^5,15–26^. Characteristics of the included studies are summarized in Table 1. Studies were predominantly at a low-to-moderate risk of bias. The median follow-up was three months and ranged from one month^19,20,22,25^ to one year^5,18^. Studies analyzed topical vancomycin vs control (2,187 patients)^5,15–20^ and topical gentamicin vs control (5,532 patients)^21–26^ as SWI prophylaxis. Patients’ baseline characteristics are reported in Table 2. Seventy two percent of patients in the vancomycin-based RAD studies were male versus 75% patients in gentamicin-based RAD studies. Median age was 58.77 in vancomycin studies vs 65.25 in gentamicin studies. Diabetes was present in 22.1% of patients in vancomycin studies and 30.4% patients in gentamicin studies. Bilateral internal mammary artery (BIMA) use was not consistently reported.

**Table 1:** Baseline characteristics of included studies.

Study**		N of pts	Topical vancomycin	Infection assessment*	Follow-up	Risk of bias
Vancomycin-based RAD	Blinding					
Basha et al.^15^	Yes	126	Paste (2.5 g powdered vancomycin mixed with 3 mL normal saline)	NR	6 months	Serious/Critical
Maldonado et al.^16^	Yes	52	Paste (1 g powdered vancomycin mixed with physiologic solution)	Chest CT at 4, 8, and 12 weeks post-operatively	12 weeks	High
Mohsin Mahmood et al.^17^	No	180	1 g vancomycin powder applied on sternal edges	NR	6 weeks	High
Pervaiz et al.^18^	Yes	276	Solution (2 g vancomycin in 50 ml normal saline)	SSWI-limited to the subcuticular & subcutaneous layers; DSWI-involved the sternal bone or wires and collections beneath the sternum,	1 year	Serious/Critical
Servito et al. [SWI]^5^	Yes	1037	Sponge soaked in 5 g vanco-mycin powder dissolved in 50 mL sterile water	Types of SWI: superficial, deep, organ space surgical site infections	1 year	Serious/Critical
Shah SJ et al.^19^	No	100	Paste (1 g vancomycin mixed with 5 ml normal saline)	NR	1 month	Serious/Critical
Vander Salm et al.^20^	No	416	Paste (1 g of powdered absorbable gelatin mixed with topical thrombin (1000 units/mL); and 250 mg powdered vancomycin	DSWI: sternal or mediastinal infections always necessitating a major operation; SSWIs: no sternal involvement	1 month	Low
Gentamycin-based RAD	Blinding		Topical gentamycin			
Bennet-Guerrero et al. [SWIPE-1]^21^	Single-blind	1502	Gentamicin-collagen sponges (total gentamicin of 260 mg) between the sternal halves at surgical closure	standardized criteria from the Centers for Disease Control and Prevention and the ASEPSIS scoring system	3 months	High
Balkanay et al.^22^	Yes	100	Solution-absorbent sponges with 320 mg of gentamicin in 250 ml of an isotonic solution	NR	1 month	High
Eklund et al.^23^	No	542	gentamicin-collagen implant (130 mg gentamicin and 280 mg collagen)	monitored daily for fever, wound discharge and other evidence of wound infection by cardiac surgeons during their hospital stay	3 months	High
Friberg et al. [LOGIP]^24^	Yes	2000	Collatamp-G sponge (280 mg collagen and 130 mg gentamicin—200 mg gentamicin sulfate)	Definitions of Centers of Disease Control and Prevention	2 months	Low
Schimmer et al.^25^	Yes	720	Sponge (2 mg gentamicin sulphate, equivalent to 1.10–1.43 mg gentamicin)	Definitions of Centers of Disease Control and Prevention	1 month	Low
Schimmer et al.^26^	NR	668	Genta-Coll resorb sponge	NR	3 month	High

CT, computed tomography; UMR, uni-and multivariable regression; PSM, propensity score matching; PRP, platelet rich plasma; DSWI, deep sternal wound infection; SSWI, superficial sternal wound infection; NR, not reported.

*El Oakley RM, Wright JE. Postoperative mediastinitis: classification and management. Ann Thorac Surg. 1996 Mar; 61(3):1030–6.

**Studies with updates; most inclusive data was considered for meta-analysis.

**Table 2:** Baseline patients’ characteristics.

Study	Intervention	Surgery	Male, %	Age, y	BMI	Diabetes, %	COPD, %	BIMA, %	NYHA III/IV	Nonelective
Vancomycin-based RAD										
Basha et al.^15^	Vancomycin	CABG	73.8	57.3	35.8	NR	16.4	NR	NR	NR
	Control		75.4	58	35.2		14.8			
Maldonado et.al.^16^	Vancomycin	NR	NR	61	27	66	NR	NR	NR	NR
	Control		NR	63	28	68				
Mohsin Mahmood et al.^17^	Vancomycin	NR	77.78	Age > 60 11.55%	25.6	20	NR	NR	NR	NR
	Control		70	Age > 60 17.78%	26.2	11.11				
Pervaiz et al.^18^	Vancomycin	CABG	74	59.1	24.8	13	NR	NR	NR	NR
	Control		77.5	62.3	25.1	22.5				
Servito et al. [SWI]^5^	Vancomycin	Mini 2.3%; Mitral 5.1%; CABG	77.34	63.61	29.9	32.6	21.5	1.3	NR	NR
	Control	Mini 2.4%; Mitral 4.5%; CABG	78.59	62.71	30.1	30.8	24.6	1.9		
Shah l. et al.^19^	Vancomycin	CABG 28%; Valve 50%; CABG + valve 4% Other 18%	60	48.34	Obesity 18%	32%	NR	NR	NR	4%
	Control	CABG 32%; Valve 48%; CABG + valve 2%; Other 18%	64	45.36	Obesity 14%	36%	NR	NR	NR	4%
Vander Salm et al.^20^	Vancomycin	CABG 75%	69	62	NR	21%	NR	NR	NR	NR
	Control	CABG 78%	67	62.5		19%				
Gentamicin-based RAD										
Bennet-Guerrero et al. [SWIPE-1]^21^	Gentamicin	CABG and/or valve repair or replacement surgery	70.4	64.2	33.1	65.5	15.5	NR	NR	0
	Control	CABG and/or valve repair or replacement surgery	70.8	64.9	32.8	68.5	14.3	NR	NR	0
Balkanay et al.^22^	Gentamicin	CABG 100%	74	56.4	NR	36	8	NR	NR	0%
	Control	CABG 100%	74	58.9	NR	30	2	NR	NR	0%
Eklund et al.^23^	Gentamicin	CABG 100%	76	64.4	27.2	22%	9%	NR	70%	0%
	Control	CABG 100%	71	64.7	27.1	23%	10%	NR	69%	0%
Friberg et al. [LOGIP]^24^	Gentamicin	CABG 74.5%, valve 13.3%, aortic aneurysm 1.1%, congenital malformation 0.6%, CABG + other 9.7%, other 0.8%	76.6	68	26.3	18.3	5.3	0.9	NR	1.1%
	Control	CABG 72.2%, valve 14.0%, aortic aneurysm 1.9%, congenital malformation 0.5%, CABG + other 10.3%, other 1.1%	76.0	68	26.6	18.0	6.0	0.4	NR	2.5%
Schimmer et al.^25^	Gentamicin	CABG 52.7%; OPCAB 8.5%; isolated valve surgery 17.8%, combination intervention 20.4%	70.5	69	28.1	28.0	14.2	NR	NR	NR
	Control	CABG 53.1%; OPCAB 7.9%; isolated valve surgery 23.4%, combination intervention 14.7%	77.4	69	28.1	32.4	13.4	NR	NR	NR
Schimmer et al.^26^	Gentamicin	CABG 100%	81.5	67.7	28.0	32.5	19.4	31.5	32.3	NR
	Control	CABG 100%	79.5	67.8	28.6	34.9	14.8	36.1	34.8	NR

Continuous variables were summarized as mean if normally distributed; non-normal distributions were summarized as median.

BMI, body mass index; COPD, chronic pulmonary obstructive disease; BIMA, bilateral internal mammary artery; NYHA, New York Heart Association; CABG, coronary artery bypass grafting; DSWI, deep sternal wound infection; OPCAB, off-pump coronary artery bypass grafting; NR, not reported.

Among vancomycin-based RAD four studies used paste^15,16,19,20^, one, vancomycin powder^17^, one, vancomycin solution^18^, and one used a sponge soaked in vancomycin^5^. All gentamicin studies evaluated gentamicin-collagen implants^23^ or sponges^21,22,24–26^ (Supplementary Table 5). Intra-venous antibiotic prophylaxis consistent mostly of second-or third -generation cephalosporins; one study reported routine iv. cefepime prophylaxis. Six studies reported on the protocol mandated blood glucose levels control^17–19,21,22,26^ (Supplementary Table 5).

### Sternal wound infections

**Figure 2:**
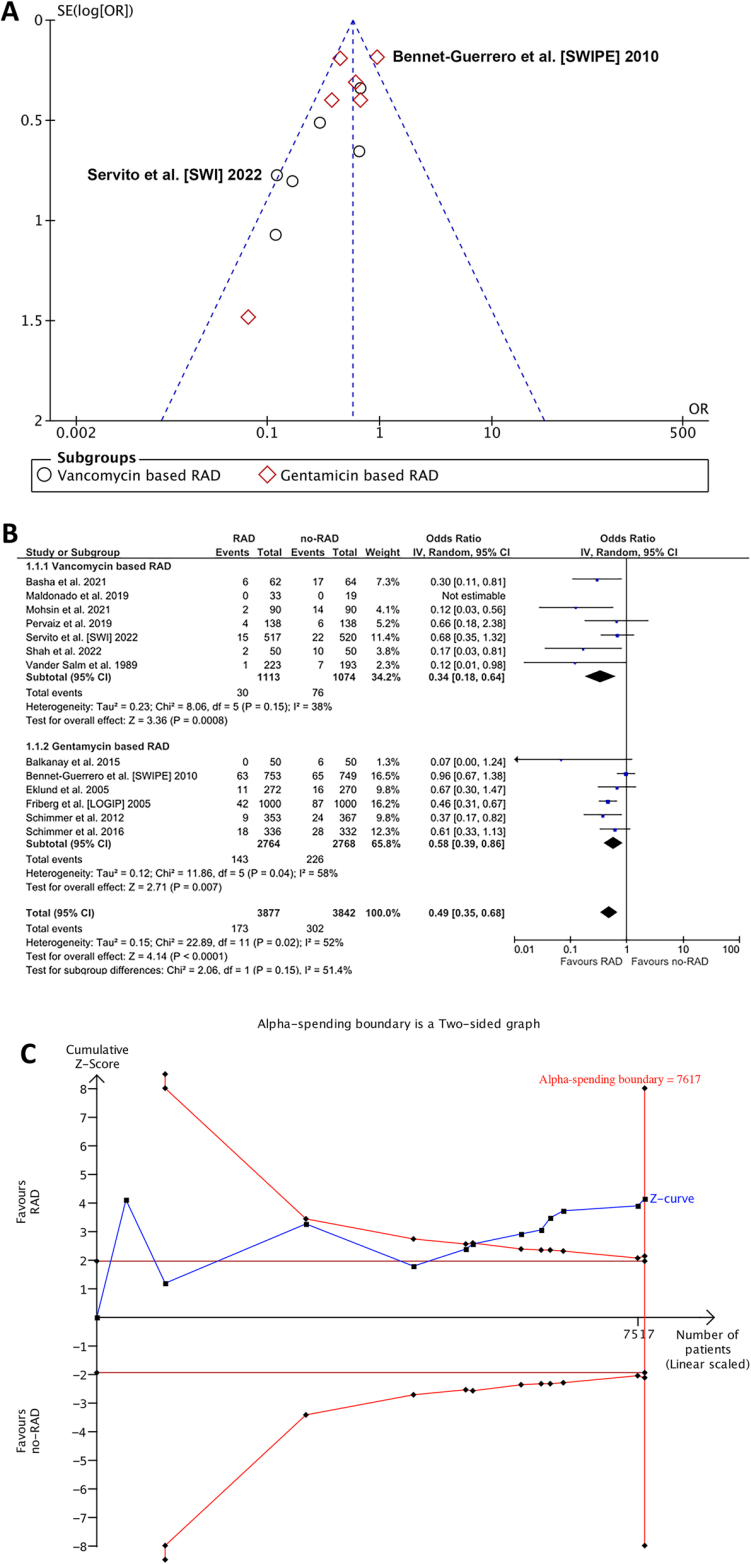
Analysis of sternal wound infections. **A** funnel plot for the assessment of publication bias; **B** individual (blue squares) and summary (black diamonds) odds ratios (ORs) along with 95% confidence intervals (CIs) and the forest plot for the comparison between RAD and no RAD; **C** Trial sequential analysis. IV, Inverse variance; CI, confidence interval; RAD, regional antibiotic delivery.

SWI definitions are available in Supplementary Table 6. All thirteen studies (7,719) contributed to the analysis of any SWI. The funnel plot for the visual assessment of publication bias is available as Fig. 2A. In the random effects model, topical antibiotic use was associated with an over 50% reduction of the odds of any SWI: OR (95% CIs): 0.49 (0.35–0.68); *p* < 0.001; *I*^2^ = 52%; that was significant regardless of the type of antibiotic (vancomycin vs. no-RAD; 0.34 (0.18–0.64); *p* < 0.001; *I*^2^ = 38% and gentamicin vs no-RAD; 0.58 (0.39–0.86); *p* = 0.007; *I*^2^ = 58%; P_heterogeneity for between subgroups comparison_ = 0.15. The corresponding rates in the overall cohort were 2.7% (30/1,113) versus 7.1% (76/1,074) for the topical vancomycin and no-vancomycin groups, and 5% (143/2,764) versus 8.2% (226/2,768) for the topical gentamicin and no-gentamicin groups (Fig. 2B).

Cumulative Z-curve passed the TSA-adjusted boundary for SWIs suggesting adequate power has been met and no further trials are needed; TSA adjusted OR was 0.49 (0.35–0.68) *P* < 0.001 (Fig. 2C).

**Figure 3:**
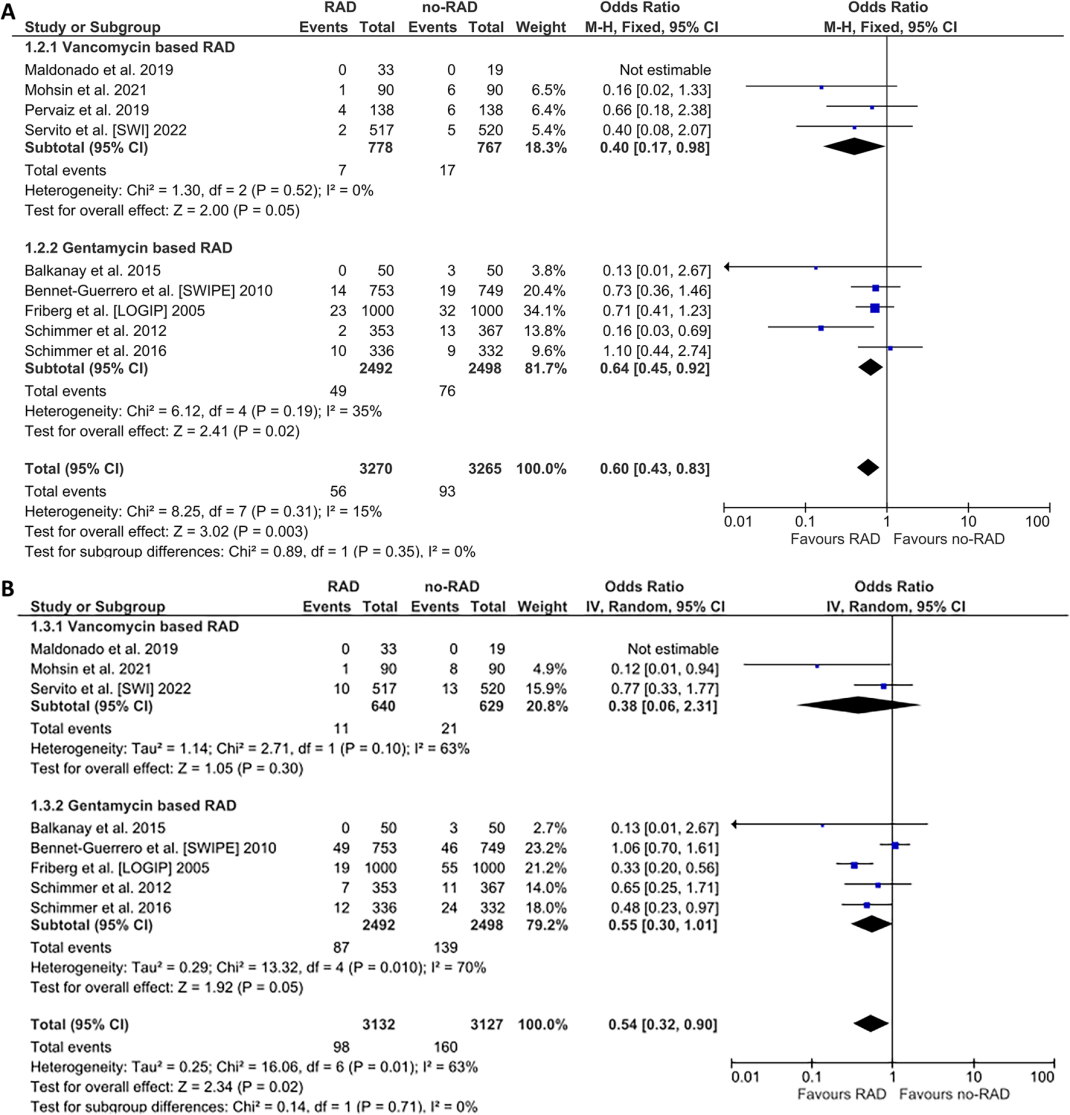
Analysis of deep sternal wound infections (**A**) and superficial sternal wound infections (**B**). Abbreviations as in Fig. 2.

Nine studies contributed to the analysis of DSWIs. The effect of topical RAD remained significant with a 40% DSWI odds reduction: OR (95%CIs): 0.60 (0.43–0.83); *p* = 0.003; *I*^2^ = 15%; The effect was similar in vancomycin trials (0.40 [0.17–0.98]; *p* = 0.05; *I*^2^ = 0%) and gentamicin studies (0.64 [0.45–0.92]; *p* = 0.02; *I*^2^ = 15%; P_heterogeneity for between subgroups comparison_ = 0.35). The corresponding rates in the overall cohort were 1.7% (56/3,270) versus 2.8% (93/3,265) for RAD and no-RAD groups respectively Fig. 3A. Superficial SWIs data were available from 8 studies: RAD was associated with a significant, over 45% reduction of the odds of SSWI: OR (95%CIs): 0.54 (0.32–0.90); *p* = 0.01; *I*^2^ = 63%; that reached borderline significance in gentamicin studies (0.55 (0.30–1.01); *p* = 0.05; *I*^2^ = 70%; P_heterogeneity for between subgroups comparison_ = 0.71. The corresponding rates in the overall cohort were 3.1 (98/3,132) versus 5.1% (160/3,127) for the RAD and no-RAD groups respectively Fig. 3B.

The incidence of mediastinitis was reported in 8 studies respectively; while numerical reduction in mediastinitis odds was seen (OR: 0.74 [0.40–1.37]) *I*^2^ = 0%; statistical significance was not reached (*P* = 0.81). Supplementary Fig. 1. No differences in mortality (OR: 0.99 [0.48–2.06]; *p* = 0.98; *I*^2^ = 5%) between RAD and no-RAD were observed. Supplementary Fig. 2.

### Systemic toxicity and microbiology

Studies did not report definition nor specific outcomes on systemic toxicity; three studies reported acute kidney injury (AKI) data; no differences between RAD and no-RAD were found: OR: 1.17 (0.57–2.40); *P* = 0.67; *I*^2^ = 75%). Four studies only reported microbiology data; the occurrence of gram-positive cultures was significantly reduced with both vancomycin and gentamicin based RAD: OR: 0.42 (0.17–1.01); *P* = 0.05; *I*^2^ = 53% and OR: 0.42 (0.27–0.64); *P* < 0.001; *I*^2^ = 40% respectively. One study only reported on the cultures of drug resistant bacteria: there were 4 and 2 cases of methicillin resistant *S. Aureus* in the gentamicin based RAD and no-RAD respectively; together with 1 case of gentamicin resistant *P. Stuarti* and 2 cases of *S. Epidermidis* in the gentamicin based RAD arm constituting total of 0.04% resistance emergence. Meta-analysis was not attempted since one study only reported the data of interest. Details on microbiology findings and systemic toxicity are available as Table 3. No differences in terms of sternal dehiscence or non-union were seen as far as RAD was concerned: Basha et al.^15^ reported 0 cases of sternal non-union in both vancomycin and control groups; SWIPE trial reports alone on patients’ feeling of chest wall instability (0 vs. 9 for gentamycin sponge and control respectively); remaining studies do not report on these data.

**Table 3:** Microbiology findings and systemic toxicity.

Study	Intervention	Microbiology	Systemic toxicity
Vancomycin-based RAD			
Basha et al.^15^	Vancomycin	NA	NA
	Control	NA	NA
Maldonado et al.^16^	Vancomycin	NA	NA
	Control	NA	NA
Mohsin Mahmood et al.^17^	Vancomycin	NA	NA
	Control	NA	NA
Pervaiz et al.^18^	Vancomycin	NA	NA
	Control	NA	NA
Servito et al. [SWI]^5^	Vancomycin	coagulase-negative Staphylococcus (4/8) Staphylococcus aureus (2/8) Other (2/8)	NA
	Control	coagulase-negative Staphylococcus (5/15) Staphylococcus aureus (4/15) Gram negative organism (2/15) Other (4/15)	NA
Shah et al.^19^	Vancomycin	NA	NA
	Control	NA	NA
Vander Salm et al.^20^	Vancomycin	S. aureus (1)	NA
	Control	S. nonaureus, Proprionobacter (1) S. nonaureus (3) S. aureus (3)	NA
Gentamicin-based RAD			
Bennet-Guerrero et al. [SWIPE-1]^21^	Gentamicin	Positive culture: 27 Acinetobacter calcoaceticus (1) Enterobacter cloacae (2) Enterococcus faecalis (1) Escherichia coli (3) Group B species streptococcus (1) Proteus mirabilis (4) Providencia stuartii (1) Pseudomonas aeruginosa (3) Serratia marcescens (2) Staphylococcus aureus (5) Staphylococcus capitis (1) Staphylococcus epidermidis (2) Streptococcus agalactiae (1)	AKI—18 (2.4%)
	Control	Positive culture: 32 Enterobacter cloacae (1) Enterococcus faecalis (1) Escherichia coli (2) Klebsiella pneumoniae (2) Klebsiella spp (1) Proteus mirabilis (5) Pseudomonas aeruginosa (3) Serratia marcescens (4) Staphylococcus aureus (6) Staphylococcus epidermidis (7) Staphylococcus hominis (1) Staphylococcus lugdunensis (1) Staphylococcus xylosus (1)	AKI—23 (3.0%)
Balkanay et al.^22^	Gentamicin	NA	AKI—0
	Control	NA	AKI—0
Eklund et al.^23^	Gentamicin	NA	NA
	Control	NA	NA
Friberg et al. [LOGIP]^24^	Gentamicin	Staphylococcus aureus (8) Coagulase-negative staphylococci (11) Other bacteria or multiple species (10) Missing or negative bacterial samples (13)	7.8%
	Control	Staphylococcus aureus (20) Coagulase-negative staphylococci (33) Gram-negative bacteria (4) Other bacteria or multiple species (15) Missing or negative bacterial samples (15)	5.1%
Schimmer et al.^25^		Detected bacteria in the 15 patients with DSWI were coagulase-negative staphylococci (68.4%), gram-negative bacteria (10.5%), Propionibacterium acnes (10.5%), and Staphylococcus aureus (5.3%) With only 2 cases of DSWI in the gentamicin group, a difference between the 2 groups could not be detected	NA
Schimmer et al.^26^	Gentamicin	NA	NA
	Control	NA	NA

RAD, regional antibiotic delivery; AKI, acute kidney injury; DSWI, deep sternal wound infection; NA, not available.

### Diabetes versus no-diabetes

**Figure 4:**
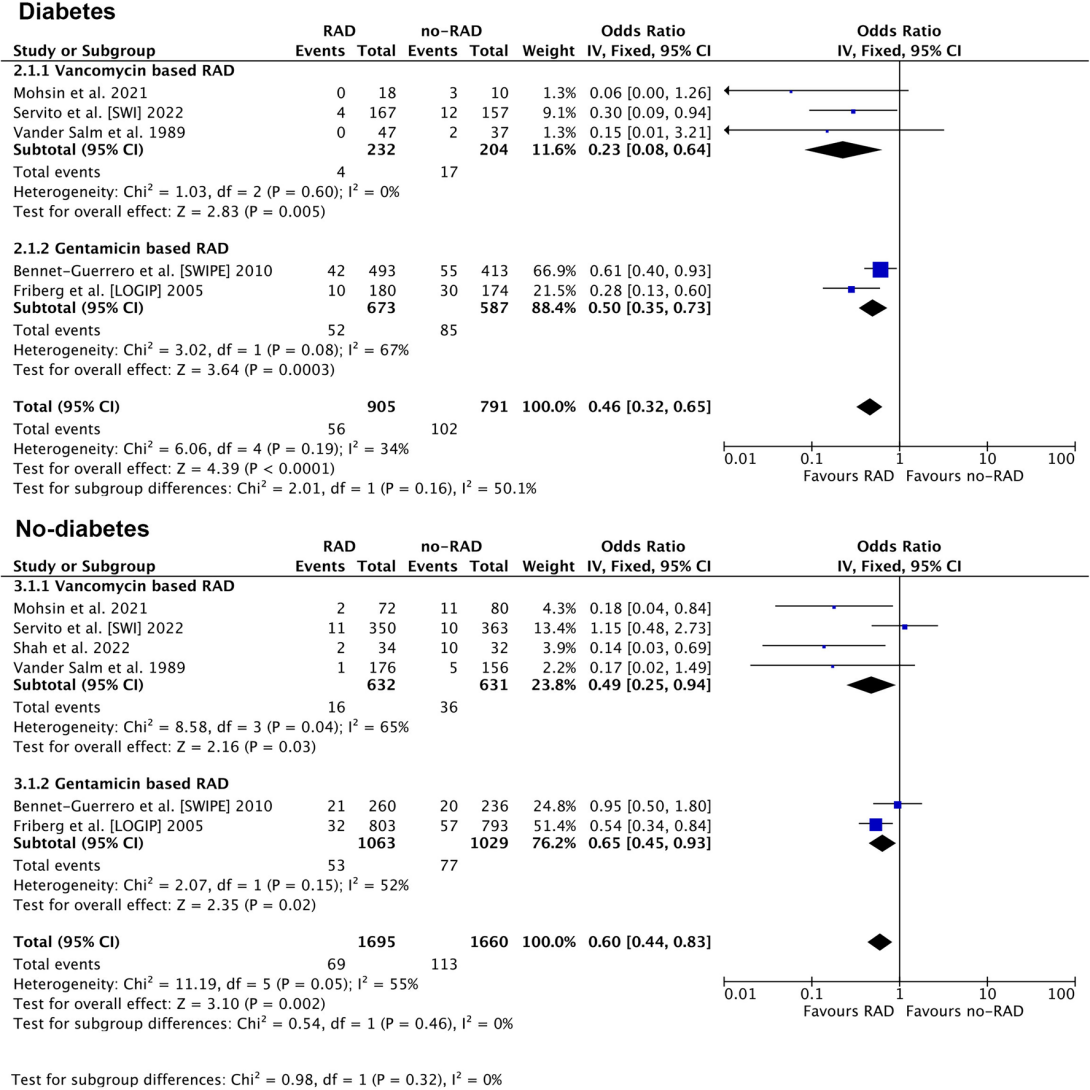
Analysis of sternal wound infections in diabetic and no-diabetic population. Abbreviations as in Fig. 2.

We observed no difference in RAD efficacy between diabetic (OR: 0.4 [0.32–0.65]; *P* < 0.001; *I*^2^ = 34%) and non-diabetic (OR: 0.60 [0.45–0.83]; *P* = 0.002; *I*^2^ = 55%) patients (*p* for subgroup difference *p* = 0.32). Figure 4.)

### Sensitivity analyses

The sensitivity analyses were consistent with the main results. Similarly, we excluded single studies, one at a time and repeated the calculations (Supplementary Table 7) and observed no significant study effect.

## Discussion

The current study is the first meta-analysis, focused on randomized controlled trials (RCTs) to address the effectiveness of two most commonly regionally administered antibiotics (RAD) in sternal wound infection (SWI) prophylaxis among patients undergoing cardiac surgeries. The main findings of this study are: (1) the odds of any SWI were significantly reduced by over 50% with any RAD; (2) RAD reduced the odds of SWI in diabetic and non-diabetic patients; (3) both antibiotics reduced the odds of cultures outside their respective serum concentrations’ activity; (4) no evidence of systemic toxicity, sternal dehiscence and resistant strains emergence was found. It is the first meta-analysis to assess jointly vancomycin and gentamicin- based protocols for RAD. Data from previous studies showed a reduction of the incidence of SWI regardless of the RAD protocol. As a result, guideline recommendations were developed, which endorsed the use of local prophylaxis together with systemic iv. antibiotics, tight glycemic control and adequate surgical techniques to control the infection rates (1). However, routine use of RAD has been avoided due to concerns of potential systemic toxicity augmented by local administration. Furthermore, there are claims of the possible emergence of bacterial strains that may develop resistance to vancomycin and gentamicin. Two recent studies^5,27^ on RAD found no benefit of local vancomycin prophylaxis in SWI reduction, which have fueled the ongoing debate.

### Rationale for local antibiotics and SSI reduction

The rationale for using local antibiotics in reducing surgical site infections (SSIs) is based on several key factors. Local antibiotics provide a targeted approach by delivering high concentrations of antimicrobial agents directly to the surgical site, effectively controlling bacteria in the vicinity of the incision. This localized application allows for higher concentrations of antimicrobial agents compared to systemic administration, enhancing their bactericidal effect and reducing bacterial growth. By minimizing systemic exposure, local antibiotics help reduce potential adverse effects and the development of antibiotic resistance. They also serve as an additional layer of prophylaxis against SSIs, inhibiting bacterial colonization at the incision site. Local antibiotics are particularly beneficial in high-risk cases and complement standard infection control practices^11^. Incorporating local antibiotics into surgical protocols can contribute to the reduction of SSIs and improve patient outcomes.

### Local antibiotic prophylaxis in cardiac surgery

The choice between gentamicin and vancomycin for RAD depends on the local antibiotic resistance patterns, specific bacteria targeted and the risk factors for infection. If the risk of gram-negative bacteria is high, such as in certain types of surgeries or patient populations, gentamicin may be preferred. If there is a higher risk of gram-positive bacteria, including MRSA, vancomycin may be more appropriate. Limited reports are available on the synergistic effects of locally applied gentamicin and vancomycin^28,29^.

### Effectiveness

Our meta-analysis found that RAD was effective prophylaxis against SWI; the odds were significantly reduced by over 50%, regardless whether vancomycin or gentamicin local prophylaxis was used.

These findings are in line with those of recent meta-analyses, which also demonstrated the benefit for single antibiotics protocols^11,30,31^.

The largest to date meta-analysis addressing vancomycin based RAD in addition to SWI incidence benefit, found that the magnitude of benefit varied across patient populations in the risk-regression analysis^11^. Patients at the highest risk of developing SWI, such as those with diabetes, reached the highest reductions of SWI as compared to controls; the magnitude of benefit from RAD in lowering SWI rates may be even greater if strict glycaemic control protocols are in place; Lazar et al. showed “0 incidence” of SSIs regardless of the baseline HbA1c levels in the previous study in which continuous insulin infusion was used to achieve tight perioperative glycemic control^2^; Furnary et al. demonstrated in a study of 5,510 patients that glycemic control rather than baseline HbA1c levels correlated with SWI incidence^32^. This was also noted in gentamicin-based RAD; where in those patients at higher risk of developing SWI such as those in whom BIMA was harvested, the magnitude of benefit was proportionally higher^30^.

The effectiveness of RAD is, beyond doubt, dependant on the local concentration of antibiotic in the wound. All the actions taken that may reduce this concentration, in turn, may result in the loss of prophylaxis against SSIs. A striking example of this phenomenon was the first large-scale RCT to address the effectiveness of gentamicin in SWI prophylaxis where the authors, in order to facilitate handling, did not follow the manufacturer’s instructions to implant the sponge; in this study, the sponge was soaked in saline prior to placing it between the sternal halves^21^, which washed away the gentamicin and resulted in no reduction in SSIs. This was later confirmed in an in-vitro study^33^. Servito et al. soaked the gauze in vancomycin solution placed it on the sternal edges at the time of surgery and then removed the gauze before rewiring the sternum at conclusion of the surgery^5^. This approach significantly reduced the concentration of the antibiotic when it was dissolved in the saline. It is, in addition, essential for the antibiotic to remain in the wound for as long as possible to act as a prophylactic agent. Similarly, single “sprinkling” of antibiotic solution over the wound as done in another study^18^ also proved to be ineffective. These flaws were explained in detail in previous reports^34–36^.

Studies to measure the effective concentrations in the wound are available from experimental studies^37^. When antibiotic wound concentrations are high and serum concentrations remain stable, it was found that the antibiotics were effective against bacteria for which systemic administration is generally not recommended^5,38^. This finding was partially confirmed in the previous meta-analysis^11^ which showed that patients who received vancomycin-based RAD and developed infections did not show an increase in vancomycin-resistant strain cultures in infected wounds. Contrarily, vancomycin non-susceptible organisms like Gram-negative strains^38^ were isolated nearly three times less frequently in the vancomycin group compared to the no-vancomycin group. This finding confirms the results of previous experimental studies. Mączyńska et al. demonstrated the in-vitro efficacy of gentamicin released from a collagen sponge carrier against Pseudomonas aeruginosa and Klebsiella pneumoniae biofilms that displayed a resistance pattern in routine diagnostics^39^. Additionally, gentamicin was shown in the in-vitro model of infected meshes used for hernia repair to prevent growth of all bacteria, including even gentamicin-resistant S. aureus strains^40^.

### Safety

The current study found no evidence of drug-resistant bacteria growth from the wounds in patients receiving RAD. Indeed, only one single study^41^ reported 6 cases of MRSA [4 in the gentamicin and 2 in the placebo arm) which together with gentamicin resistant strains (3 vs. 3 in gentamicin and no-gentamicin arms) constituted 0.04% emergence of resistant bacteria when compared to roughly 8% infection rate in the RAD control arm. In addition, an often-raised concern regarding the widespread use antibiotics in general, is its presumed association with an increase in drug resistant strains. It is, however, persistent systemic exposure to sub-inhibitory levels of vancomycin or gentamicin that may cause resistant strains. The development of vancomycin intermediate-resistant Staphylococcus was demonstrated in an in vitro model with persistent vancomycin exposure above 10 mg/L while the emergence of vancomycin resistance has not been reported in studies on the use of topical vancomycin^42^. Furthermore, extended intravenous prophylaxis or long-term intravenous antibiotic administration may be associated with systemic toxicity. Although none of the studies included in our analysis specifically investigated systemic toxicity, we conducted an analysis of acute kidney injury (AKI) as a potential surrogate endpoint. Similar to our previous findings, we observed no significant differences between patients who received RAD and those who did not in terms of AKI incidence. Application of vancomycin paste or gentamicin sponges did not impair the wound healing process. One histopathological study revealed that gentamicin was highly effective in reducing infection and promoting callus repair, resulting in early bone healing^43^. Vancomycin paste, in contrast to wax that will hardly be absorbed and produces a foreign body giant cell reaction, is perfectly water-soluble. Limited data was available for the analysis of sternal dehiscence or non-union.

This systematic review and meta-analysis included only 13 studies, number which may have resulted in type I errors due to an increased risk of random errors resulting from sparse studies and data. To gauge the risk of type I errors, we utilized TSA, a method integrating estimated information size (accumulated sample size of incorporated trials) with an adjusted threshold for statistical significance in cumulative meta-analyses. If the cumulative Z-curve intersects the trial sequential monitoring boundary or enters the futility area, it suggests that there might be adequate evidence for the expected intervention effect, and additional trials may not be necessary. Conversely, when the evidence is considered insufficient to draw a conclusion, additional trials are needed to confirm the results. In conducting this TSA, we estimated the required information size using α = 0.05 (two sided) and β = 0.20 (power = 80%) and a relative risk reduction of 20% in outcomes. The cumulative Z-curve surpassed the TSA-adjusted boundary for SWIs indicating that sufficient power has already been reached and robust benefit of RAD in reducing SWIs is well-established, and there is no need for further trials.

### Limitations

We must acknowledge several limitations to the current meta-analysis. First, the absence of a standardized prophylaxis protocol across the included studies resulted in varying rates of surgical wound infections (SWIs) in the control groups, contributing to substantial observed heterogeneity. The assessment using ROB analysis indicated a high risk of bias in several studies, although our sensitivity analysis, which excluded those studies, confirmed the consistency of the overall results. Furthermore, there was limited reporting on information regarding off-pump techniques, BIMA use and harvesting techniques, which are known factors that can also influence the occurrence of SWIs. Data on patients with higher risk of opportunistic infections, such as on chronic glucocorticoids or with concomitant hematological diseases was also limited. Data on systemic antibiotic levels were unavailable for other studies in our analysis, limiting our ability to evaluate redosing strategies and the impact of intravenous antibiotics on outcomes.

## Conclusions

The results of this systematic review and updated meta-analysis confirm the high effectiveness of the topical antibiotics vancomycin and gentamicin, in preventing sternal wound infections after cardiac surgery without compromising safety and with no signs of side effects including systemic toxicity and emergence of resistant bacteria.

### Supplementary Information

Supplementary Information.

## Data Availability

The datasets used and analysed during the current study are available from the corresponding author on reasonable request.
