# Author Correction: Regional antibiotic delivery for sternal wound infection prophylaxis a systematic review and meta-analysis of randomized controlled trials

**DOI:** 10.1038/s41598-024-66983-1

**Published:** 2024-07-15

**Authors:** Mariusz Kowalewski, Michalina M. Kołodziejczak, Tomasz Urbanowicz, Maria Elena De Piero, Silvia Mariani, Michał Pasierski, Maged Makhoul, Maria Comanici, Emil Julian Dąbrowski, Matteo Matteucci, Giulio Massimi, Radosław Litwinowicz, Adam Kowalówka, Wojciech Wańha, Federica Jiritano, Gennaro Martucci, Giuseppe Maria Raffa, Pietro Giorgio Malvindi, Łukasz Kuźma, Piotr Suwalski, Roberto Lorusso, Paolo Meani, Harold Lazar, Thoracic Research Centre, Jakub Brączkowski, Dario Fina, Mirosław Gozdek, Giovanni Chiarini, Federica Jiritano, Michalina M. Kołodziejczak, Adam Kowalówka, Mariusz Kowalewski, Łukasz Kuźma, Roberto Lorusso, Radosław Litwinowicz, Tong Li, Giuseppe Marchese, Gennaro Martucci, Giulio Massimi, Matteo Matteucci, Maged Makhoul, Pietro Giorgio Malvindi, Silvia Mariani, Paolo Meani, Anna Olasińska, Michał Pasierski, Luigi Pannone, Maria Elena De Piero, Giuseppe Maria Raffa, Sebastian Stec, Jakub Staromłyński, Serena Todaro, Tomasz Urbanowicz, Wojciech Wańha

**Affiliations:** 1 Clinical Department of Cardiac Surgery and Transplantology, National Medical Institute of the Ministry of Interior and Administration, Wołoska 137, 02-507 Warsaw, Poland; 2 Cardio-Thoracic Surgery Department, Heart and Vascular Centre, Maastricht University Medical Centre, Cardiovascular Research Institute Maastricht (CARIM), Maastricht, The Netherlands; 3 Department of Anaesthesiology and Intensive Care, Antoni Jurasz University Hospital No. 1, Collegium Medicum Nicolaus Copernicus University, Bydgoszcz, Poland; 4 Cardiac Surgery and Transplantology Department, Poznań University of Medical Sciences, Poznan, Poland; 5 Department of Cardiac Surgery, Harefield Hospital, London, UK; 6 Department of Invasive Cardiology, Medical University of Bialystok, Bialystok, Poland; 7 Cardiac Surgery Unit, Department of Medicine and Surgery, ASST dei Sette Laghi, University of Insubria, Varese, Italy; 8 Cardiac Surgery Unit, Santa Maria della Misericordia Hospital, Perugia, Italy; 9 Department of Cardiac Surgery, Regional Specialist Hospital, Grudziądz, Poland; 10 Department of Cardiac Surgery, Faculty of Medical Sciences, Upper-Silesian Heart Center, Medical University of Silesia, Katowice, Poland; 11 Department of Invasive Cardiology, School of Medicine in Katowice, Medical University of Silesia, Katowice, Poland; 12 Department of Experimental and Clinical Medicine, Magna Graecia University, Catanzaro, Italy; 13 Department of Anesthesia and Intensive Care, Istituto Mediterraneo Per i trapianti e Terapie ad alta specializzazione (IRCCS-ISMETT), Palermo, Italy; 14 Department for the Treatment and Study of Cardiothoracic Diseases and Cardiothoracic Transplantation, IRCCS-ISMETT, Palermo, Italy; 15 Cardiac Surgery Unit, Lancisi Cardiovascular Center, Ospedali Riuniti Delle Marche, Polytechnic University of Marche, Ancona, Italy; 16 Department of Cardiothoracic and Vascular Anesthesia and Intensive Care Unit, IRCCS Policlinico, San Donato Milanese, Milan, Italy; 17 Boston University School of Medicine, Boston, MA USA; 18 Thoracic Research Centre, Innovative Medical Forum, Collegium Medicum Nicolaus Copernicus University, Bydgoszcz, Poland; 19 Department of Cardiology, Città di Lecce Hospital, GVM Care and Research, Lecce, Italy; 20 Department of Cardiology, Hospital of the Ministry of Interior, Bydgoszcz, Poland; 21 Division of Anesthesiology, Intensive Care and Emergency Medicine, University of Brescia, Brescia, Italy; 22 Department of Experimental and Clinical Medicine, “Magna Graecia” University of Catanzaro, Catanzaro, Italy; 23 Department of Anesthesiology and Intensive Care, Collegium Medicum Bydgoszcz, Antoni Jurasz University Hospital No.1, Nicolaus Copernicus University Torun, 85-094 Bydgoszcz, Poland; 24 Department of Cardiac Surgery, School of Medicine in Katowice, Medical University of Silesia, Katowice, Poland; 25 Department of Cardiac Surgery, Upper-Silesian Heart Center, Katowice, Poland; 26 Cardiovascular Research Institute Maastricht (CARIM), Maastricht, The Netherlands; 27 CAROL - Cardiothoracic Anatomy Research Operative Lab, Department of Cardiovascular Surgery and Transplantology, Institute of Cardiology, Jagiellonian University Medical College, Krakow, Poland; 28 Department of Cardiothoracic, Transplantation and Vascular Surgery, Hannover Medical School, Hannover, Germany; 29 Department of Pathophysiology and Transplantation, Università degli Studi di Milano, Milan, Italy; 30 Department of Anesthesia and Intensive Care, Istituto Mediterraneo per i Trapianti e Terapie ad alta Specializzazione (IRCCS-ISMETT), UPMCI (University of Pittsburgh Medical Center Italy), Palermo, Italy; 31 Department of Medicine and Surgery, Circolo Hospital, University of Insubria, Varese, Italy; 32 Heart Rhythm Management Centre, Postgraduate Program in Cardiac Electrophysiology and Pacing, European Reference Networks Guard-Heart, Universitair Ziekenhuis Brussel - Vrije Universiteit Brussel, Brussels, Belgium; 33 Division of Electrophysiology, Cardioneuroablation, Catheter Ablation and Cardiac Stimulation, Subcarpathian Center for Cardiovascular Intervention, Sanok, Poland; 34 Department of Cardiology and Structural Heart Diseases, Medical University of Silesia, Katowice, Poland

Correction to: *Scientific Reports* 10.1038/s41598-024-60242-z, published online 27 April 2024

In the original version of this Article Giuseppe Maria Raffa and Gennaro Martucci were incorrectly affiliated with ‘Thoracic Research Centre, Innovative Medical Forum, Collegium Medicum Nicolaus Copernicus University, Bydgoszcz, Poland’. The correct affiliations are listed below.

Giuseppe Maria Raffa:

Department for the Treatment and Study of Cardiothoracic Diseases and Cardiothoracic Transplantation, IRCCS-ISMETT, Palermo, Italy

Gennaro Martucci:

Department of Anesthesia and Intensive Care, Istituto Mediterraneo Per i trapianti e Terapie ad alta specializzazione (IRCCS-ISMETT), Palermo, Italy.

The original Article has been corrected.

