# Supplementary material for: Regional antibiotic delivery for sternal wound infection prophylaxis a systematic review and meta-analysis of randomized controlled trials
Source: Sci Rep. 2024 Apr 27;14:9690. doi: 10.1038/s41598-024-60242-z (PMC11055886; doi:10.1038/s41598-024-60242-z)
Supplement: Supplementary file 1 — Supplementary Information. [file 41598_2024_60242_MOESM1_ESM.docx]

**Regional Antibiotic Delivery for Sternal Wound Infection Prophylaxis. Meta-Analysis of Randomized Controlled Trials.**

**Supplementary material**

**Table of contents:**

**Tables:**

**Supplementary Table 1.** Preferred Reporting Items for Systematic reviews and Meta-Analyses (PRISMA) 2020 Checklist.

**Supplementary Table 2.** Search Strategy.

**Supplementary Table 3.** Grading of Recommendations Assessment, Development and Evaluations (GRADE) analysis for randomized controlled trials.

**Supplementary Table 4.** ROB risk of bias analysis in the randomized controlled trials.

**Supplementary Table 5.** Antibiotic background prophylaxis, RAD characteristics and glycaemic control.

**Supplementary Table 6.** Sternal wound infection definitions.

**Supplementary Table 7.** Sensitivity analysis. Analysis repeated for the primary endpoint by deleting each single study, one at a time, and repeating the calculations.

**Figures:**
**Supplementary Figure 1.** Analysis of mediastinitis incidence in RAD and no-RAD subgroup. analysis. IV, Inverse variance; CI, confidence interval; RAD, regional antibiotic delivery

**Supplementary Figure 2.** Analysis of morality in RAD and no-RAD subgroup. Abbreviations as in Supplementary Figure 1.

**References to excluded studies**

**Supplementary Table 1.** Preferred Reporting Items for Systematic reviews and Meta-Analyses (PRISMA) 2020 Checklist

| **Section and Topic** | **Item #** | **Checklist item** | **Location where**  **item is reported** |
| --- | --- | --- | --- |
| **TITLE** | | |  |
| Title | 1 | Identify the report as a systematic review. | 1 |
| **ABSTRACT** | | |  |
| Abstract | 2 | See the PRISMA 2020 for Abstracts checklist. | 2 |
| **INTRODUCTION** | | |  |
| Rationale | 3 | Describe the rationale for the review in the context of existing knowledge. | 4 |
| Objectives | 4 | Provide an explicit statement of the objective(s) or question(s) the review addresses. | 4 |
| **METHODS** | | |  |
| Eligibility criteria | 5 | Specify the inclusion and exclusion criteria for the review and how studies were grouped for the syntheses. | 5-7 |
| Information sources | 6 | Specify all databases, registers, websites, organisations, reference lists and other sources searched or consulted to identify studies. Specify the date when each source was last searched or consulted. | 5-7 |
| Search strategy | 7 | Present the full search strategies for all databases, registers and websites, including any filters and limits used. | 5-7 |
| Selection process | 8 | Specify the methods used to decide whether a study met the inclusion criteria of the review, including how many reviewers screened each record and each report retrieved, whether they worked independently, and if applicable, details of automation tools used in the process. | 5-7 |
| Data collection process | 9 | Specify the methods used to collect data from reports, including how many reviewers collected data from each report, whether they worked independently, any processes for obtaining or confirming data from study investigators, and if applicable, details of automation tools used in the process. | 5-7 |
| Data items | 10a | List and define all outcomes for which data were sought. Specify whether all results that were compatible with each outcome domain in each study were sought (e.g. for all measures, time points, analyses), and if not, the methods used to decide which results to collect. | 5-7, Supplementary Table 2 |
|  | 10b | List and define all other variables for which data were sought (e.g. participant and intervention characteristics, funding sources). Describe any assumptions made about any missing or unclear information. | 5-6, Supplementary Table 2 |
| Study risk of bias assessment | 11 | Specify the methods used to assess risk of bias in the included studies, including details of the tool(s) used, how many reviewers assessed each study and whether they worked independently, and if applicable, details of automation tools used in the process. | 5-7 |
| Effect measures | 12 | Specify for each outcome the effect measure(s) (e.g. risk ratio, mean difference) used in the synthesis or presentation of results. | 7 |
| Synthesis methods | 13a | Describe the processes used to decide which studies were eligible for each synthesis (e.g. tabulating the study intervention characteristics and comparing against the planned groups for each synthesis (item #5)). | 5-7 |
|  | 13b | Describe any methods required to prepare the data for presentation or synthesis, such as handling of missing summary statistics, or data conversions. | 5-7 |
|  | 13c | Describe any methods used to tabulate or visually display results of individual studies and syntheses. | 5-7 |

|  |  |  |  |
| --- | --- | --- | --- |
|  | 13d | Describe any methods used to synthesize results and provide a rationale for the choice(s). If meta-analysis was performed, describe the model(s), method(s) to identify the presence and extent of statistical heterogeneity, and software package(s) used. | 5-7 |
|  | 13e | Describe any methods used to explore possible causes of heterogeneity among study results (e.g. subgroup analysis, metaregression). | 5-7 |
|  | 13f | Describe any sensitivity analyses conducted to assess robustness of the synthesized results. | 5-7 |
| Reporting bias assessment | 14 | Describe any methods used to assess risk of bias due to missing results in a synthesis (arising from reporting biases). | 5-7 |
| Certainty assessment | 15 | Describe any methods used to assess certainty (or confidence) in the body of evidence for an outcome. | 5-7 |
| **RESULTS** | | |  |
| Study selection | 16a | Describe the results of the search and selection process, from the number of records identified in the search to the number of studies included in the review, ideally using a flow diagram. | 5-7, Figure 1 |
|  | 16b | Cite studies that might appear to meet the inclusion criteria, but which were excluded, and explain why they were excluded. | Supplementary Referenecs |
| Study characteristics | 17 | Cite each included study and present its characteristics. | Tables 1-3, References |
| Risk of bias in studies | 18 | Present assessments of risk of bias for each included study. | 5-7, Supplementary Table 4 |
| Results of individual studies | 19 | For all outcomes, present, for each study: (a) summary statistics for each group (where appropriate) and (b) an effect estimate and its precision (e.g. confidence/credible interval), ideally using structured tables or plots. | Figures 2A, 2B, 2C, 3A, 3B, 4 |
| Results of syntheses | 20a | For each synthesis, briefly summarise the characteristics and risk of bias among contributing studies. | Table 1 |
|  | 20b | Present results of all statistical syntheses conducted. If meta-analysis was done, present for each the summary estimate and its precision (e.g. confidence/credible interval) and measures of statistical heterogeneity. If comparing groups, describe the direction of the effect. | 8-10 |
|  | 20c | Present results of all investigations of possible causes of heterogeneity among study results. | 8-10 |
|  | 20d | Present results of all sensitivity analyses conducted to assess the robustness of the synthesized results. | Supplementary Table 7 |
| Reporting biases | 21 | Present assessments of risk of bias due to missing results (arising from reporting biases) for each synthesis assessed. | Supplementary Table 4 |
| Certainty of evidence | 22 | Present assessments of certainty (or confidence) in the body of evidence for each outcome assessed. | Supplementary Table 3 |
| **DISCUSSION** | | |  |
| Discussion | 23a | Provide a general interpretation of the results in the context of other evidence. | 11 |
|  | 23b | Discuss any limitations of the evidence included in the review. | 15 |
|  | 23c | Discuss any limitations of the review processes used. | 15 |
|  | 23d | Discuss implications of the results for practice, policy, and future research. | 11-15 |
| **OTHER INFORMATION** | | |  |
| Registration and protocol | 24a | Provide registration information for the review, including register name and registration number, or state that the review was not registered. | 6 |
|  | 24b | Indicate where the review protocol can be accessed, or state that a protocol was not prepared. | 15 |
|  | 24c | Describe and explain any amendments to information provided at registration or in the protocol. | N/A |
| Support | 25 | Describe sources of financial or non-financial support for the review, and the role of the funders or sponsors in the review. | 16 |
| Competing interests | 26 | Declare any competing interests of review authors. | 16 |
| Availability of data, code and other materials | 27 | Report which of the following are publicly available and where they can be found: template data collection forms; data extracted from included studies; data used for all analyses; analytic code; any other materials used in the review. | 16 |

*From:*  Page MJ, McKenzie JE, Bossuyt PM, Boutron I, Hoffmann TC, Mulrow CD, et al. The PRISMA 2020 statement: an updated guideline for reporting systematic reviews.

BMJ 2021;372:n71. doi: 10.1136/bmj.n71

**Supplementary Table 2.** Search strategy.

PubMed/MEDLINE

| No. | Search | Results |
| --- | --- | --- |
| 1. | Vancom* paste [Title/Abstract] OR Vancom* gel [Title/Abstract] OR Vancom* slurry [Title/Abstract] OR Vancom* ointment [Title/Abstract] | 1,203 |
| 2. | (Vancom* [Title/Abstract] AND (local [Title/Abstract] OR topical [Title/Abstract]) | 1,918 |
| 3. | Stern* [Title/Abstract] OR Mediastinitis [Title/Abstract] | 48,143 |
| 4. | (#1 OR #2) AND #3 | 39 |
| 5. | Gentam* paste [Title/Abstract] OR gentam* gel [Title/Abstract] OR gentam* slurry [Title/Abstract] OR gentam* ointment [Title/Abstract] | 1,060 |
| 6. | (Gentam* [Title/Abstract] AND (local [Title/Abstract] OR topical [Title/Abstract]) | 2,073 |
| 7. | (#5 OR #6) AND #3 | 32 |

EMBASE

| No. | Search | Results |
| --- | --- | --- |
| 1. | Vancom* paste/exp OR Vancom* gel/exp OR Vancom* slurry/exp OR Vancom* ointment/exp OR (Vancom* paste OR Vancom* gel OR Vancom* slurry OR Vancom* ointment):ti:,ab | 172 |
| 2. | Vancom*:ti,ab AND (local OR topical):ti,ab | 2,731 |
| 3 | Stern*:ti,ab OR mediastinitis:ti,ab | 73 |
| 4. | (#1 OR #2) AND #3 | 2,744 |
| 5. | Gentam* paste/exp OR Gentam* gel/exp OR Gentam* slurry/exp OR Gentam* ointment/exp OR (Gentam* paste OR Gentam* gel OR Gentam* slurry OR Gentam* ointment):ti:,ab | 172 |
| 6. | Gentam*:ti,ab AND (local OR topical):ti,ab | 2,731 |
| 7. | (#5 OR #6) AND #3 | 2,744 |

**Supplementary Table 3.** Grading of Recommendations Assessment, Development and Evaluations (GRADE) analysis for randomized controlled trials.

| **Number of studies** | **Certainty assessment** | | | |  |  | **Effect** |  | **Quality** |
| --- | --- | --- | --- | --- | --- | --- | --- | --- | --- |
|  | **Risk of bias** | **Inconsistency** | **Indirectness** | **Imprecision** | **Other considerations** | **Control** | **Topical vancomycin** | **Risk Ratio  (95% CI)** |  |
| *Sternal wound infection* | | | | |  |  |  |  |  |
| vancomycin (7) | Not serious | Not serious | Not serious | Not Serious | None | 76 (1,074) | 30 (1,113) | 0.38 (0.22-0.67) | High |
| gentamycin (6) | Not serious | Not serious | Not serious | Not Serious | None | 226 (2,768) | 143 (2,764) | 0.63 (0.41-0.87) | High |
| *Superficial sternal wound infection* | | | | |  |  |  |  |  |
| vancomycin (3) | Not serious | Not serious | Not serious | Not Serious | None | 21 (629) | 11 (640) | 0.40 (0.07-2.31) | High |
| gentamycin (5) | Not serious | Not serious | Not serious | Not Serious | None | 139 (2,498) | 87 (2,492) | 0.57 (0.31-1.02) | High |
| *Deep sternal wound infection* | | | | |  |  |  |  |  |
| vancomycin (5) | Not serious | Not serious | Not serious | Not Serious | None | 17 (767) | 7 (778) | 0.41 (0.17-0.99) | High |
| gentamycin (5) | Not serious | Not serious | Not serious | Not Serious | None | 76 (2,498) | 49 (2,492) | 0.65 (0.45-0.92) | High |
| *Mediastinitis* | | | | |  |  |  |  |  |
| vancomycin (5) | Not Serious | Not serious | Not serious | Serious | None | 3 (464) | 1 (506) | 0.41 (0.06-2.81) | Moderate |
| gentamycin (3) | Not Serious | Not serious | Not serious | Serious | None | 20 (1,320) | 16 (1,322) | 0.80 (0.42-1.53) | Moderate |
| *Mortality* | | | | |  |  |  |  |  |
| vancomycin (2) | Not serious | Not serious | Not serious | Not serious | None | 3 (69) | 1 (83) | 0.33 (0.04-3.10) | Low |
| gentamycin (2) | Not serious | Not serious | Not serious | Not serious | None | 17 (1,050) | 19 (1,050) | 1.12 (0.58-2.14) | Low |

CI, confidence interval.

**Supplementary Table 4.** ROB risk of bias analysis in the randomized controlled trials

| **Study** | **Randomization** | **Deviation** | **Missing data** | **Outcome assessment** | **Selective reporting** | **Overall** |
| --- | --- | --- | --- | --- | --- | --- |
| ***Vancomycin-based RAD*** | | | | | | |
| Basha MAA et al. 2021 [15] | Low risk | Low risk | Low risk | Some concerns | Low risk | Some concerns |
| Maldonado LAM et al. 2019 [16] | Low risk | Low risk | Low risk | High risk | Low risk | High risk |
| Mohsin Mahmood M et al. 2021 [17] | Some concerns | Low risk | Low risk | High risk | Low risk | High risk |
| Pervaiz F et al. 2019 [18] | Some concerns | Low risk | Low risk | Low risk | Low risk | Some concerns |
| Servito M et al. [SWI] 2022 [5] | Low risk | Low risk | Some concerns | Low risk | Some concerns | Some concerns |
| Shah SJ et al. 2022 [19] | Low risk | Low risk | Low risk | Some concerns | Low risk | Some concerns |
| Vander Salm TJ et al. 1989 [20] | Low risk | Low risk | Low risk | Low risk | Low risk | Low risk |
| ***Gentamycin-based RAD*** | | | | | | |
| Bennet-Guerrero E. et al. [SWIPE-1] 2010 [21] | Low risk | Low risk | Low risk | Low risk | Low risk | Low risk |
| Balkanay OO et al. 2015 [22] | Low risk | Low risk | Low risk | Some concerns | Low risk | Some concerns |
| Eklund AM et al. 2005 [23] | Low risk | Low risk | Some concerns | Some concerns | Low risk | Some concerns |
| Friberg O et al. [LOGIP] 2005 [24] | Low risk | Low risk | Low risk | Low risk | Low risk | Low risk |
| Schimmer C et al. 2012 [25] | Low risk | Low risk | Low risk | Some concerns | Low risk | Some concerns |
| Schimmer C et al. 2016 [26] | Low risk | Low risk | Low risk | Some concerns | Low risk | Some concerns |

RAD, regional antibiotic delivery

**Supplementary Table 5.** Antibiotic background prophylaxis and topical vancomycin characteristics.

| **Study** | **Staphylococcal screening/**  **mupirocine eradication** | **Background IV antibiotic prophylaxis** | **Antibiotic composition** | | **Antibiotic handling** | **Glycaemic control** |
| --- | --- | --- | --- | --- | --- | --- |
| ***Vancomycin-based RAD*** | |  |  | |  |  |
|  | Basha MAA et al. 2021 [15] | NR | Within 60 minutes before the skin incision: 50 mg/kg. A 2nd dose of 1g cefepime given if the surgical incision remained open for more than 3–4 hours. Subsequent 2g cefepime daily for 48 hours postoperatively. | Paste (2.5g powdered vancomycin mixed with 3 mL normal saline) | applied to both sternal halves | NR |
|  | Maldonado LAM et al. 2019 [16] | NR | NR | Paste (1g powdered vancomycin mixed with physiologic solution) | Topical vancomycin mass in the spongy tissue exposed by surgical sternotomy, prior to sternal closure | NR |
|  | Mohsin Mahmood M et al. 2021 [17] | NR | Ceftriaxone (1g) and co-amoxiclav (1.2g) given at induction and continued for 72 hours postoperatively. | 1g vancomycin powder | injection vancomycin powder is applied on sternal edges | Injection insulin regular used according to blood glucose levels to keep it within normal limits |
|  | Pervaiz F et al. 2019 [18] | NR | Cefazolin 2 g IV every 8h and vancomycin (1 g IV every 12 hours) on induction of anaesthesia. and continued for 48 hours after surgery | Solution (2g vancomycin in 50 ml normal saline) | Vancomycin solution sprayed in the sternal wound before closure. | IV insulin infusions were used in diabetic patients starting at the time of induction of anesthetic and continuing for 24 hours to maintain serum glucose values between 120 and 180 mg/dL. |
|  | Servito M et al. [SWI] 2022 [5] | NR | IV antibiotics given immediately before skin incision in accordance with local standards. | 5g vancomycin in 50mL sterile water. | Sponge soaked in vancomycin applied on the sternum during the operation. | NR |
|  | Shah SJ et al. 2022 [19] | NR | 1g ceftriaxone IV on induction and continued after surgery. | Paste (1g vancomycin mixed with 5ml normal saline) | Applied on the sternal edges. | Careful glycemic control for the diabetic patients according to guidelines to keep blood sugar less than 180 mg/dL. |
|  | Vander Salm TJ et al. 1989 [20] | NR | Cefazolin; vancomycin if penicillin allergy; continued for 36 h after surgery | Paste (1 g of powdered absorbable gelatine mixed with topical thrombin (1000 units/mL); and 250 mg powdered vancomycin. | Applied on the sternal edges. | NR |
| ***Gentamycin-based RAD*** | |  |  | |  |  |
|  | Bennet-Guerrero E. et al. [SWIPE-1] 2010 [21] | Allowed but not required | Cefazolin or cefuroxime initiated 60 minutes prior to skin incision. Vancomycin was administered to individuals who were allergic to cephalosporin or penicillin, or those at increased risk of methicillinresistant Staphylococcus aureus colonization. It was permissible for ciprofloxacin to be added to vancomycin if greater gram-negative coverage was desired. Dosing was weight based and was to be continued for at least 24 hours, but not more than 48 hours | 100 cm^2^ (5x20 cm) sponge contained 280 mg of collagen and 130 mg of gentamicin | Two sponges inserted between the sternal halves along the full length of the sternum immediately before closure of the sternum | Insulin administered in operating room plus first 24h postoperatively. |
|  | Balkanay OO et al. 2015 [22] | NR | Intravenous prophylactic antibiotics were given to all of the patients, with cefazolin being administered to 98 patients (98%) and vancomycin to two patients (2%) with a beta (b)-lactam antibiotic allergy. | solvent for the gentamicin group included a total of 320 mg of gentamicin in 250 ml of an isotonic solution | sponges beneath the edges of the sternum retractor, and these were in contact with all layers of the skin, subcutaneous tissues, and both sides of the sternum | Fifteen patients required an insulin perfusion protocol to control their blood glucose levels in the early postoperative period, |
|  | Eklund AM et al. 2005 [23] | NR | two doses of intravenous (IV) cefuroxime 1.5 g in 6 h. The patients that were hospitalized at least three days pre-operatively also received IV vancomycin 500 mg on two occasions (cardiac surgery in case of acquisition of hospital pathogens) | gentamicin-collagen implant (Gentacoll) which contains 130 mg gentamicin and 280 mg collagen | Sponge was implanted underneath the sternum before wound closure | NR |
|  | Friberg O et al. [LOGIP] 2005 [24] | NR | Antibiotic prophylaxis was given IV, in accordance with the protocol of each center, starting immediately before the skin incision and then with 1 to 2 repetitions during surgery (cloxacillin at one center and dicloxacillin at the other, at the doses 2 g and 1 g, respectively), continued every 8th hour for 24 to 48 hours postoperatively, depending on whether the drainage tubes had been removed. | Collatamp-G (a flat absorbable bovine collagen sponge with gentamicin sulfate. A 10x10x0.5 cm sponge contains 280 mg collagen and 130 mg gentamicin (200 mg gentamicin sulfate). | Two sponges in the wound immediately before closure, the sponges were cut into appropriate sizes and put between the sternal halves. | NR |
|  | Schimmer C et al. 2012 [25] | NR | Intravenous cefuroxime (1.5 g twice per day) starting 30 minutes before the operation for up to 48 hours postoperatively | gentamicin-collagen sponges (also containing 2 mg gentamicin sulphate, equivalent to 1.10–1.43 mg gentamicin; verum) | Sponge was implanted retrosternally without premoistening | NR |
|  | Schimmer C et al. 2016 [26] | No routine | A second generation cephalosporin (cefuroxime). The first dose was given in the OR 30–60 min before skin incision and then continued for 24– 48 h. | The Genta-Coll resorb sponge | was inserted between and under the sternal halves during sternum osteosynthesis at the end of the operation | Blood glucose levels monitored frequently and insulin administered if necessary to achieve blood glucose levels <200 mg/dL. Postoperatively, blood glucose levels monitored every hour in the first 24 h on the ICU and blood glucose level over 200 mg/dL not allowed. |

CPB, cardiopulmonary bypass; IV, intravenous; NR, not reported; RAD, regional antibiotic delivery.

**Supplementary Table 6.** Sternal wound infection definition.

| **Study** | | **Sternal wound infection definition** |
| --- | --- | --- |
| ***Vancomycin-based RAD*** | | |
|  | Basha MAA et al. 2021 [15] | NR |
|  | Maldonado LAM et al. 2019 [16] | NR |
|  | Mohsin Mahmood M et al. 2021 [17] | NR |
|  | Pervaiz F et al. 2019 [18] | Depths 1 and 2 were defined as a superficial infectious process limited to the subcuticular and subcutaneous layers with no involvement of the sternal bone. Depths 3 and 4, which involved the sternal bone or wires and collections beneath the sternum, were considered deep infections. A wound was considered infected only if a positive culture for an organism was obtained. Reported infections included all infections that developed within 1 year of surgery |
|  | Servito M et al. [SWI] 2022 [5] | NR |
|  | Shah SJ et al. 2022 [19] | NR |
|  | Vander Salm TJ et al. 1989 [20] | DSWI: sternal or mediastinal infections always necessitating a major operation; SSWIs: no sternal involvement. |
| ***Gentamycin-based RAD*** | | |
|  | Bennet-Guerrero E. et al. [SWIPE-1] 2010 [21] | Standardized criteria including those from the Centers for Disease Control and Prevention and the ASEPSIS scoring system. |
|  | Balkanay OO et al. 2015 [22] | NR |
|  | Eklund AM et al. 2005 [23] | Assessment of surgicalsite infections (SSIs) was made according to the criteria published by the Centers for Disease Control in 1992. |
|  | Friberg O et al. [LOGIP] 2005 [24] | Criteria for definition and classification of surgical site infections according to Centers of Disease Control and Prevention were used with minor modifications |
|  | Schimmer C et al. 2012 [25] | DSWI and SSWI were defined on the basis of the criteria of the US Centers for Disease Control and Prevention (a positive bacterial culture from mediastinal tissue or fluid, and an intraoperative clinical picture of mediastinitis in the presence of one of the following: chest pain, sternal instability, fever greater than 38ºC, purulent secretion from the mediastinum, or positive bacterial culture from the blood or mediastinal drainage fluid).  An SSWI was deemed present if there was microbiological evidence of microbes in the cutaneous or subcutaneous tissue at the site of the incision, the incision was purposely reopened, or at least one of the following secondary diagnoses was made: purulent secretion from the superficial incision with or without laboratory confirmation, positive bacterial culture from a fluid or tissue sample taken from the superficial incision under aseptic conditions, and at least 1 sign/symptom of infection: redness, heat, pain, or swelling. |
|  | Schimmer C et al. 2016 [26] | NR |

DSWI, deep sternal wound infection; SSWI, superficial sternal wound infection; NR, not reported;RAD, regional antibiotic delivery.

**Supplementary Table 7.** Sensitivity analysis. Analysis repeated for the primary endpoint by deleting each single study, one at a time, and repeating the calculations.

| **Study** | | **Point estimates after study removal** | | | |  |
| --- | --- | --- | --- | --- | --- | --- |
|  |  | **subgroup RAD** | | **total** | |  |
| ***Vancomycin-based RAD*** | | | |  | |  |
|  | Basha MAA et al. 2021 [15] | | 0.32 [0.14-0.74] | | 0.50 [0.35-0.72] | |
|  | Maldonado LAM et al. 2019 [16] | | 0.34 [0.18-0.64] | | 0.49 [0.34-0.68] | |
|  | Mohsin Mahmood M et al. 2021 [17] | | 0.41 [0.22-0.75] | | 0.52 [0.38-0.73] | |
|  | Pervaiz F et al. 2019 [18] | | 0.28 [0.13-0.60] | | 0.57 [0.33-0.68] | |
|  | Servito M et al. [SWI] 2022 [5] | | 0.26 [0.14-0.48] | | 0.46 [0.31-0.67] | |
|  | Shah SJ et al. 2022 [19] | | 0.37 [0.18-0.73] | | 0.51 [0.36-0.72] | |
|  | Vander Salm TJ et al. 1989 [20] | | 0.37 [0.19-0.71] | | 0.51 [0.36-0.71] | |
| ***Gentamycin-based RAD*** | | | |  | |  |
|  | Bennet-Guerrero E. et al. [SWIPE-1] 2010 [21] | | 0.49 [0.37-0.65] | | 0.45 [0.34-0.60] | |
|  | Balkanay OO et al. 2015 [22] | | 0.61 [0.42-0.88] | | 0.50 [0.36-0.70] | |
|  | Eklund AM et al. 2005 [23] | | 0.56 [0.35-0.89] | | 0.46 [0.32-0.67] | |
|  | Friberg O et al. [LOGIP] 2005 [24] | | 0.63 [0.40-0.99] | | 0.47 [0.32-0.71] | |
|  | Schimmer C et al. 2012 [25] | | 0.63 [0.41-0.96] | | 0.50 [0.34-0.72] | |
|  | Schimmer C et al. 2016 [26] | | 0.56 [0.34-0.92] | | 0.46 [0.31-0.68] | |

RAD, regional antibiotic delivery

**Supplementary Figure 1.**


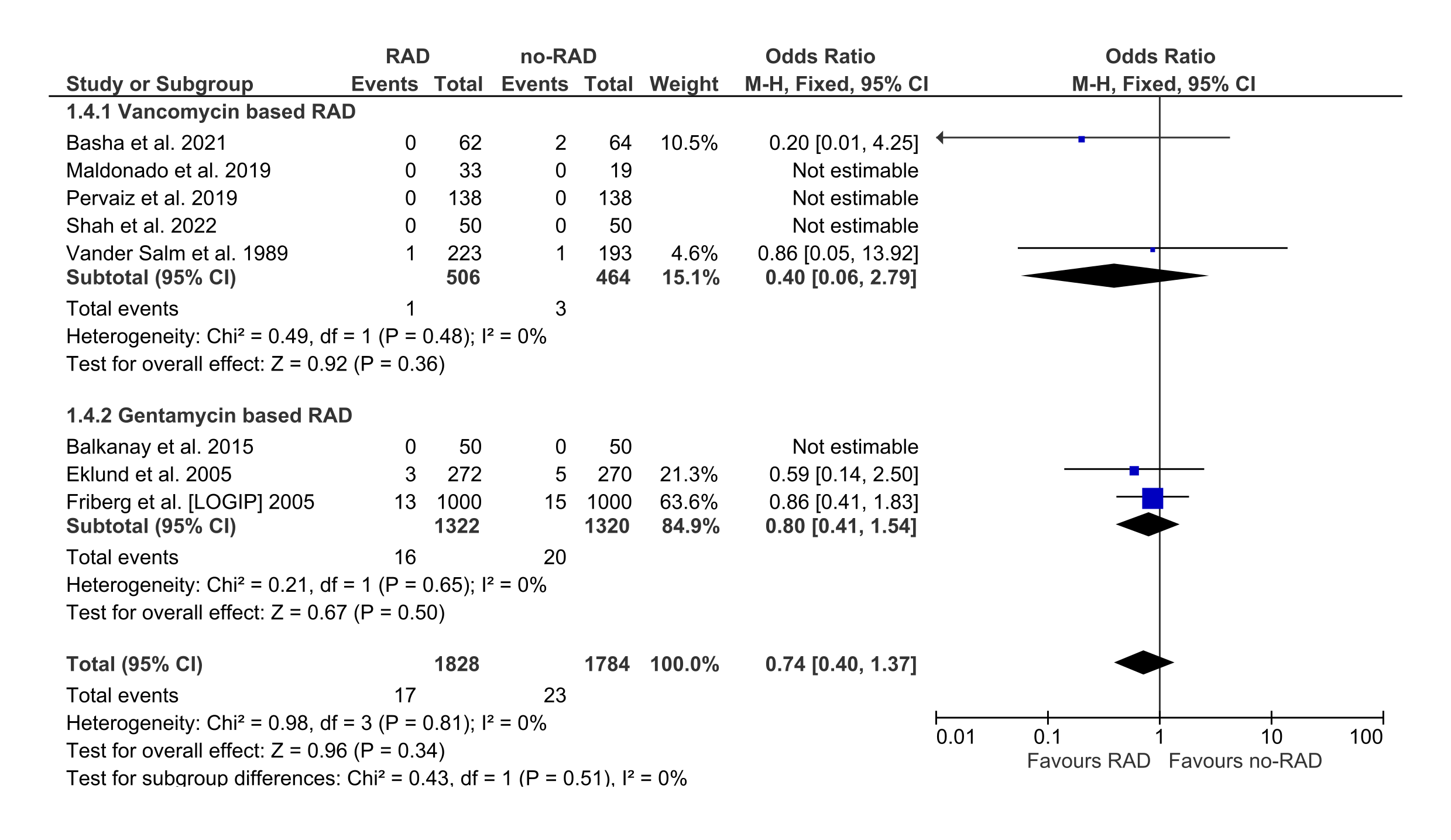


**Supplementary Figure 2.**


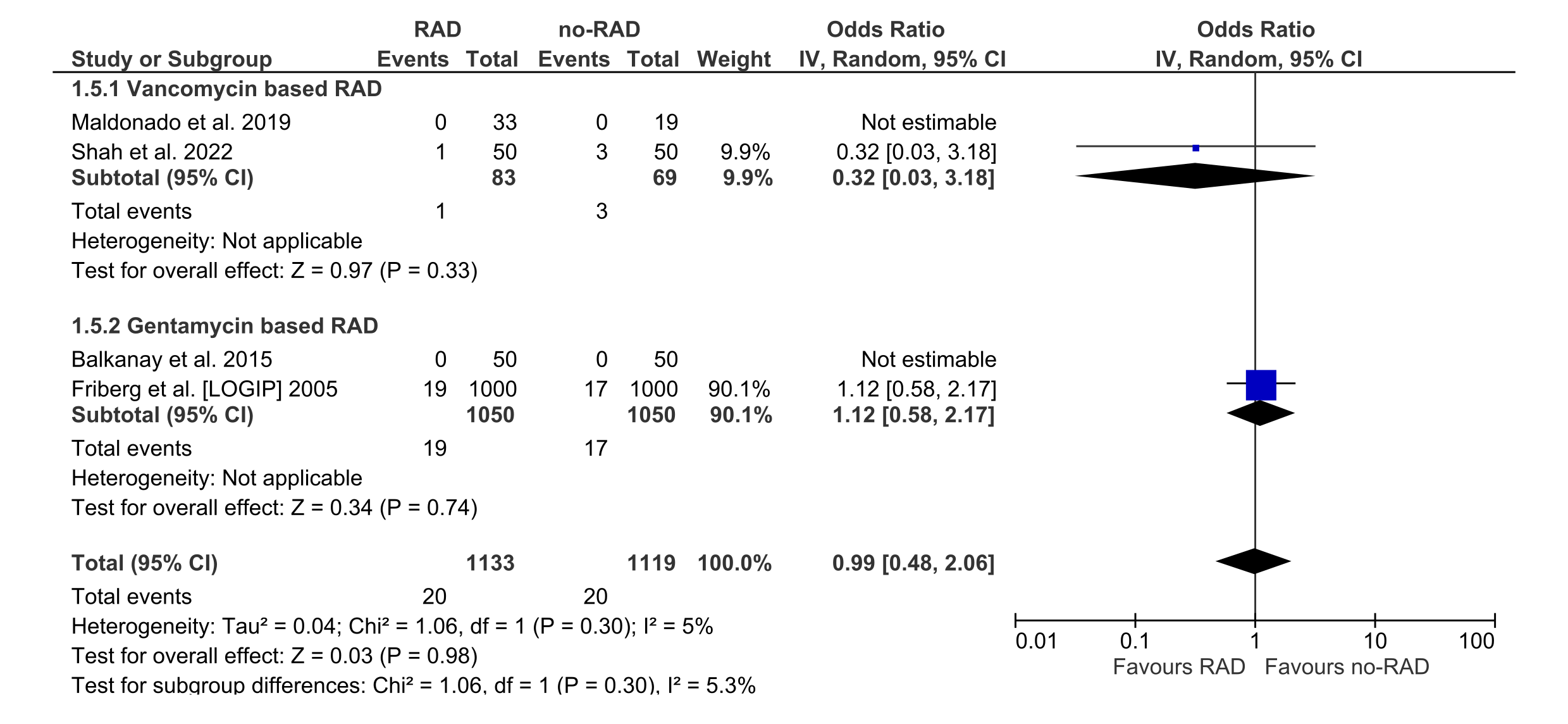


**References to excluded studies**

1. Dhadwal K, Al-Ruzzeh S, Athanasiou T, et al (2007) Comparison of clinical and economic outcomes of two antibiotic prophylaxis regimens for sternal wound infection in high-risk patients following coronary artery bypass grafting surgery: a prospective randomised double-blind controlled trial. Heart 93:1126–1133. https://doi.org/10.1136/HRT.2006.103002

2. Vos RJ, Van Putte BP, De Mol BAJM, et al (2022) Application of local gentamicin in the treatment of deep sternal wound infection: a randomized controlled trial. Eur J Cardiothorac Surg 61:1135–1141. https://doi.org/10.1093/EJCTS/EZAB479

3. Hafermann MJ, Kiser TH, Lyda C, et al (2014) Weight-based versus set dosing of vancomycin for coronary artery bypass grafting or aortic valve surgery. J Thorac Cardiovasc Surg 147:1925–1930. https://doi.org/10.1016/J.JTCVS.2013.12.037
